# Adequacy of calcium and vitamin D nutritional status in a nationally representative sample of Irish teenagers aged 13–18 years

**DOI:** 10.1007/s00394-022-02939-3

**Published:** 2022-07-03

**Authors:** Kevin D. Cashman, Laura Kehoe, John Kearney, Breige McNulty, Janette Walton, Albert Flynn

**Affiliations:** 1 Cork Centre for Vitamin D and Nutrition Research, School of Food and Nutritional Sciences, and Department of Medicine, University College Cork, Cork, Ireland; 2 School of Food and Nutritional Sciences, University College Cork, Cork, Ireland; 3 School of Biological and Health Sciences, Technological University Dublin, Dublin, Ireland; 4 Institute of Food and Health, School of Agriculture and Food Science, Science Centre - South, University College Dublin, Belfield, Dublin 4, Ireland; 5 Department of Biological Sciences, Munster Technological University, Cork, T12 P928 Ireland

**Keywords:** Teenagers, National survey, Vitamin D status, Serum 25-hydroxyvitamin D, Calcium intake, Vitamin D intake

## Abstract

**Context and purpose:**

In light of the key roles of vitamin D and calcium in adolescent bone health, there is a critical need for representative data on nutritional status for both micronutrients in teenagers. The present work used data from the recent representative *National Teens’ Food Survey II* (2019–2020) to assess calcium and vitamin D intakes of teenagers in Ireland, including adequacy of such intakes, as well as, for the first time, to characterise serum 25-hydroxyvitamin D (25(OH)D) concentrations and their determinants.

**Methods:**

Usual calcium and vitamin D intake estimates were generated using food intake data (via 4-day weighed food records) from a nationally representative sample of teenagers aged 13–18 years in Ireland (*n* 428). Serum 25(OH)D was measured (via LC–MS/MS) in the 57.5% (*n* 246) who provided a blood sample.

**Results:**

Sixty-seven and 94% of Irish teenagers had intakes of calcium and vitamin D below the respective Estimated Average Requirements values, reflecting a high degree of inadequacy of intake for both micronutrients (and higher in girls than boys; *P* < 0.001). In addition, 21.7% and 33.1% of teenagers had serum 25(OH)D < 30 nmol/L (risk of vitamin D deficiency) and 30–49.9 nmol/L (inadequacy), respectively. Extended winter sampling, being aged 16–18 years, low total vitamin D intake, being overweight/obese or being of non-white skin type were significant (*P* < 0.05) predictors of serum 25(OH)D < 30 nmol/L.

**Conclusions:**

There was a high prevalence of inadequacy of intake of calcium and vitamin D in Irish teenagers, and a fifth were at increased risk of vitamin D deficiency.

## Introduction

Osteoporosis is a global health problem, and is estimated to cause around 9 million fractures annually, with Europe and the Americas accounting for half [1]. Low bone mineral mass is the main factor underlying osteoporotic fracture [2], and it depends on the peak bone mass (PBM) achieved during childhood and adolescence as well as the rate of subsequent age-related bone loss [3]. Therefore, optimization of lifestyle factors known to influence PBM is an important strategy aimed at reducing risk of osteoporosis or low bone mass later in life [4, 5]. The US National Osteoporosis Foundation (NOF)’s position statement on modifiable lifestyle factors that can influence the development of PBM highlighted that amongst the nutrition-related factors, calcium and vitamin D received a Grade A and B, respectively, within their strength of available evidence grading system [5]. This adjudged strong and moderate evidence in relation to calcium and vitamin D, respectively, is also reflected in several other important reports from authorities in the US [6–8], Europe [9, 10], and more globally [11, 12].

A working group of experts, set up by the European Commission with the aim of devising strategies for prevention of osteoporosis, recommended that policies be developed and implemented to advise the general public and health professionals about calcium and vitamin D nutrition, based on agreed recommended intakes, at all stages of life [9]. A key component of this is the critical data collection on calcium intake as well as vitamin D intake and status, especially representative population- or population subgroup-level data [9, 13]. This was highlighted as an early and important step in the roadmap for action for prevention of vitamin D deficiency, as proposed by another international expert working group on the topic [13]. Of note, by life stage, recommended intakes for calcium on either side of the Atlantic are highest for adolescents [7, 14]. The Institute of Medicine (IOM) in the US has indicated that in addition to assessment of dietary intake of vitamin D relative to the recommended intake, consideration also needs to be given to serum 25-hydroxyvitamin D (25(OH)D) concentration, as the most useful integrated marker of vitamin D exposure [7].

A recent review of child and adolescent nutrient intakes from current national dietary surveys of European populations highlighted that only a third (*n* 18) of the 53 countries of the WHO European region had nutrient intake information [15]. Only 15 of these surveys reported intake data for calcium in adolescents [15]. Likewise, only 14 the surveys reported intake data for vitamin D in adolescents [15], and a *PUBMED* search of these showed that the vast majority of surveys did not have serum 25(OH)D data [16]. In Ireland, the nationally representative *National Teens’ Food Survey* (2005–2006) reported mean calcium intakes of 1070 and 738 mg/day for boys and girls aged 13–17 years, respectively [17]. Benchmarking these intakes against the EU Average Requirement (960 mg/d) and US Estimated Average Requirement (1100 mg/day) values for calcium [7, 14] highlights a significant degree of inadequacy of intake amongst Irish adolescents, especially girls. The mean vitamin D intakes for Irish boys and girls aged 13–17 years were 3.7 and 2.7 μg/day, respectively [18], with 97 and 98%, respectively, having intakes below the US Estimated Average Requirement (EAR) value for vitamin D of 10 μg/day [7]. In the EU, an EAR for vitamin D could not be established based on available evidence [10]. Unfortunately, the survey did not include blood sampling and thus no serum 25(OH)D data are available to benchmark against suggested thresholds of deficiency/adequacy. In the UK, data from the rolling *National Diet and Nutrition Survey* (NDNS) (2008/9–2018/19) suggest that those aged 11–18 years had a prevalence of serum 25(OH)D < 25 nmol/L of 18.4%, on average over the period, which was higher than that observed for children (4–10 years), adults (18–64 years) and older adults (≥ 65 years) at 7.9, 16.9 and 14.0%, respectively [19].

The recent *National Teens’ Food Survey II* (NTFS II), conducted in 2019–2020 [20], assessed dietary intake of 13–18 year-old teenagers in the Republic of Ireland (*n* 428) and in addition secured blood samples from willing participants (*n* 246). Thus, the objectives of the present work were to use data from this nationally representative sample of teenagers in the Republic of Ireland to assess their calcium and vitamin D intakes and compare these against the respective EAR values to estimate the adequacy of intakes of these important bone-related micronutrients, and also to identify key dietary sources contributing to these intakes. In addition, in the case of vitamin D, serum 25(OH)D was measured and concentrations compared against internationally-suggested thresholds of deficiency and inadequacy. Finally, we examined determinants of serum 25(OH)D and predictors of vitamin D deficiency.

## Subjects and methods

The NTFS II was a cross-sectional study that was carried out by the Irish Universities Nutrition Alliance (IUNA), an alliance of the nutrition centres at academic institutions in Ireland, including University College Dublin, Munster Technological University, University College Cork and Technological University Dublin (www.iuna.net). Teenagers aged 13–18 years (212 boys, 216 girls) representative of this age group in the population were selected from post-primary schools throughout the Republic of Ireland. A detailed description of the methodology used in the NTFS II is available elsewhere [20]. However, an overview of salient aspects of subject sampling and recruitment procedures, as well as methods of data collection and laboratory analysis pertinent to the objectives of the present work, are outlined in the following sections.

### Ethical approval

The study was approved by the Clinical Research Ethics Committee of the Cork Teaching Hospitals and the Human Ethics Research Committee of University College Dublin (Ref: ECM 4 (II) 04/12/18 and ECM 3 (c) 15/04/19). The study was conducted according to the guidelines laid down in the Declaration of Helsinki. Written informed consent was obtained from all teenage participants and their parent(s)/guardian(s).

### Sampling procedure, inclusion /exclusion criteria and sample recruitment

The fieldwork for the survey was carried out from March 2019 to March 2020, ensuring a seasonal balance to the data and biological sample collection. To achieve a nationally representative sample of teenagers aged 13–18 years, a database of 772 post-primary schools in Ireland, available from the Department of Education and Skills, was used. Schools were selected to provide a demographically balanced sample with respect to urban versus rural location and socio-economic grouping. In brief, an introductory letter and information about the survey was posted to the principal of each selected school. This was followed up by a phone call from the coordinating nutritionist. If the principal agreed to his/her school’s participation in the survey, a suitable date and time for the coordinating nutritionist to visit the school was arranged. Seventy three percent of schools selected agreed to participate in the survey. The school principal was given information packs to give to students (randomly selected from the school roll) to bring home to their parent(s)/guardian(s). Information packs contained an introductory letter, an information brochure and a reply slip. If the parent/guardian and the selected teenager were interested in finding out more about participating in the survey, they were instructed to fill out their contact details on the reply slip and return it to the school. Teenagers who returned a reply slip were excluded if they were not between the ages of 13 and 18 years (inclusive), if they belonged to an age-group, sex or geographical location category for which the appropriate number of teenagers had already been recruited or if another member of their household had already been recruited for participation in the survey. The coordinating nutritionist in each centre contacted the parent(s)/guardian(s) of all eligible teenagers who returned a reply slip and if they agreed to participate in the survey, a suitable time and date for a fieldworker to visit was arranged. All fieldworkers were qualified nutritionists.

The overall response rate for the survey was 57%. Demographic analysis of the sample showed it to be representative of teenagers in Ireland with respect to sex and urban/rural divide when compared to Census 2016 data [21]. However, the sample contained a higher proportion of teenagers of professional workers and a lower proportion of teenagers of semi-skilled and unskilled workers than the national population and all data in this study have been weighted to account for these differences. Of the total group of 428 respondents, 57.5% (*n* 246) provided a blood sample.

### Food intake data collection, food quantification and estimation of nutrient intake

The teenage participants were asked to record detailed information (at brand level) regarding the amount and types of all foods, beverages and supplements consumed over four consecutive days (including at least one weekend day) in a food diary that was provided. Participants were also provided with a digital food scale and asked to weigh as many foods and beverages as possible, including leftovers. A quantification protocol that had been established by the IUNA for its’ previous surveys [22] was also used for the NTFS II. Eighty-eight percent of foods consumed were weighed directly or assigned a manufacturer’s weight. Where foods were not weighed, researchers used an age-appropriate photographic food atlas, standard portion sizes and household measures at subsequent visits to aid with quantifying the amount of food consumed [20]. Participants were encouraged to keep all food, beverage and nutritional supplement packaging to provide further detail on the foods consumed.

Nutrient intakes were estimated from food intakes using the UK food composition database [23]. During the survey, modifications were made to include recipes of composite dishes, nutritional supplements, fortified foods and generic Irish foods that were commonly consumed and may not be reflected in the UK food composition database. Furthermore, as the UK food composition database does not contain values for some potentially important sources of vitamin D, an approach previously outlined by Black et al*.* [18] was used to update these values for vitamin D using data from the USDA National Nutrient Database [24] for white fish, smoked salmon, processed meat (including ham) and mushrooms, and the Danish Food Composition Database [25] for whole, semi-skimmed and skimmed milk.

Total calcium intake and total vitamin D intake (representing intake from diet plus supplement use), which will be referred to as usual intakes hereafter, were estimated using the validated National Cancer Institute (NCI) Method using SAS Enterprise Guide^©^ [26] (date of download: July 2015). The prevalence of inadequate intakes of vitamin D and calcium was estimated using EAR values from the US IOM for vitamin D (10 µg/days) and calcium (1100 mg/days) [7] as well as from the European Food Safety Authority (EFSA) for calcium (13–17 years: 960 mg/days, 18 years: 860 mg/days) [14]. As under-reporting of food consumption can result in an overestimate of the prevalence of inadequacy in a population group, under-reporters (URs) were identified (46% of total sample) and excluded from these analyses only using Goldberg’s cut-off 2 criterion updated by Black (which evaluates the ratio of energy intake to basal metabolic rate (EI:BMR) against age-specific energy cut offs based on physical activity levels) [27–30]. The risk of excessive intakes of vitamin D and calcium was evaluated using the Tolerable Upper Intake Level (UL) values from both the US IOM [7] and EFSA [31] for vitamin D (100 μg/days), and from the US IOM [7] in the case of calcium (3000 mg/days). The mean percent contribution of 19 pre-defined food groups to calcium and vitamin D intakes was estimated using the mean proportion method by determining the mean daily intake (MDI) of the nutrient per participant from each particular food group and dividing this value by the participant’s MDI of the nutrient from all foods [32]. The mean proportion method provides information about the sources that are contributing to the nutrient intake ‘per person’ and is the preferred method when determining important food sources of a nutrient for individuals in the population group as opposed to investigating the sources of a nutrient within the food supply.

### Participant questionnaires and anthropometry

Teenage participants and their parent(s)/guardian(s) were asked to complete questionnaires with regard to the teenager on general health and lifestyle (including skin colour and sun holiday practices), on determinants of food choice and eating behaviours as well as for physical activity levels. Full details of these questionnaires are presented elsewhere [20].

Weight and height were measured for each teenager, as described in detail elsewhere [20]. Body Mass Index (BMI), calculated by weight (kg) divided by height squared (m^2^), was used to indirectly assess adiposity. Age-and-sex-specific BMI charts were used to determine the prevalence of overweight and obesity in this sample of Irish teenagers. Due to the absence of age- and sex-specific BMI charts for an Irish reference population, the International Obesity Task Force (IOTF) age-and-sex-specific BMI cut-offs for defining overweight and obesity between 2 and 18 years were used so that international comparisons could be made [33].

### Blood collection and analysis of serum 25-hydroxyvitamin D

Teenage participants were asked to provide a fasting blood sample to assess their nutritional status and metabolic indicators of health. The samples were collected by a qualified phlebotomist at designated centres within the survey area as described elsewhere [20]. Blood was processed to serum, aliquots of which were then stored at − 80 °C until required for analysis.

Serum concentrations of total 25(OH)D (i.e. 25(OH)D_2_ plus 25(OH)D_3_) were measured by the *Cork Centre for Vitamin D and Nutrition Research* at University College Cork using a liquid chromatography–tandem mass spectrometry method, described in detail elsewhere [34], and applying the Vitamin D Standardization Program (VDSP) protocols [35]. These protocols have been developed with the goal of promoting a measurement of 25(OH)D that is accurate and comparable over time, location, and laboratory procedure to improve clinical and public health practice worldwide [36]. Serum 25(OH)D data have been standardized from a number of nationally representative surveys, including that of Irish adults [37]. The intra-assay CV of the method was < 5% for all 25(OH)D metabolites, while the inter-assay CV was < 6%.

Standardized serum 25(OH)D concentrations were compared with cut-offs for 25(OH)D as per the US IOM Dietary Reference Intake committee’s definitions: persons are at risk of deficiency at serum 25(OH)D concentrations < 30 nmol/L, whereas persons are at risk of vitamin D inadequacy at serum 25(OH)D concentrations between 30 and 49.9 nmol/L [7]. In addition, a serum 25(OH)D concentration < 25 nmol/L has also been a traditional cut-off used in the UK to define vitamin D deficiency on the basis of metabolic bone disease [38] and thus was also included.

### Statistical analysis

Statistical analysis of the data were conducted using IBM^©^ SPSS^©^ Version 26.0 (SPSS, Inc., Chicago, IL, USA). Usual calcium intake and usual vitamin D intake as well as BMI were not normally distributed and, therefore, values were logarithmically transformed prior to statistical analysis, to achieve near-normal distributions. Tests for independence were used to compare demographic variables such as age-group, sex, season of sampling, and vitamin D-containing supplement use between the subset of NTFS II teenagers with a serum 25(OH)D measurement value (*n* 246) and the complete NTFS II dataset (*n* 428). Differences in height, weight, BMI, and usual vitamin D and calcium intake between the two groups were examined using unpaired Student’s *t* tests. Differences in dietary vitamin D and calcium between sexes and age-groups were examined using unpaired Student’s *t* tests. Differences in the proportion of teenagers with vitamin D and calcium intakes below the EAR between sexes were assessed by *χ*^2^ tests.

Two-way ANOVA was used to investigate sex, age-group and their interaction on serum 25(OH)D concentrations. Three-way ANOVA was used to investigate sex, age-group, and season as well as their interactions on serum 25(OH)D concentrations.

Multiple linear regression analysis was performed to identify independent predictors of serum 25(OH)D. The following categorical variables were included: season (coded as: 0, extended winter, November–March; 1, extended summer, April–October); sex (coded as: 0, female; 1, male); age-grouping (coded as: 0, aged 13–15 years; 1, aged 16–18 years); taking a sun holiday (coded as: 0, no; 1, yes); time spent outdoors during daylight hours (coded as 1, none; 2, less than 15 min; 3, 15–30 min; 4, 30–60 min; 5, 1–2 h; 6, 3–4 h; 7, > 4 h); and skin colour (coded as: 0, white; 1, non-white). The continuous numerical variables, BMI (kg/m^2^), usual vitamin D intake (μg/d), and usual calcium intake (mg/d) were also included. The analysis was also performed with use of vitamin D-containing supplements (coded as: 0, no; 1, yes) and vitamin D intake from diet only (μg/d) in place of usual vitamin D intake, which reflects total intake of vitamin D from all sources. Unstandardized beta (B) values with their associated standard errors (SE) and *P*-values are presented. The B and SE for any logarithmically transformed determinant which was found to be significant within the model, was presented as based on the non-transformed data for ease of presentation and interpretation.

Multiple logistic regression was used to investigate predictors of risk of vitamin D deficiency (serum 25 (OH)D < 30 nmol/l), whilst adjusting for possible confounding factors. The following categorical variables were included: season (coded as: 0, extended winter, November–March; 1, extended summer, April–October); sex (coded as: 0, female; 1, male); age-grouping (coded as: 0, aged 13–15 years; 1, aged 16–18 years); taking a sun holiday (coded as: 0, no; 1, yes); being overweight or obese (coded as: 0, no; 1, yes); skin colour (coded as: 0, white; 1, non-white); usual vitamin D intake above or below the median (coded as: 0, greater than median; 1, less than median); usual calcium intake above or below the median (coded as: 0, greater than median; 1, less than median). *P* values < 0.05 were considered statistically significant.

## Results

### Baseline characteristics of the teenagers

The NTFS II is a nationally representative sample of teenagers in the Republic of Ireland. Characteristics of the participants who provided a blood sample within the NTFS II (*n* 246) were compared with those of all survey participants (*n* 428) (Table 1). No significant differences (*P* > 0.05, in all cases) were evident between the two groups (Table 1).

**Table 1:** Characteristics of the National Teens' Food Survey-II participants – all (*n* 428) and the subset with a serum 25-hydroxyvitamin D measurement value (*n* 246)*

Characteristics	All survey participants *%*	Participants with a S-25(OH)D *%*
Sex		
Boys	49.5	50.0
Girls	50.5	50.5
Age-group (years)		
13–15	55.1	48.4
16–18	44.9	51.6
Season of sampling^a^		
Extended winter	54.2	56.5
Extended summer	45.8	43.5
Vitamin D-containing supplement use	7.5	8.1

	Median	IQR	Median	IQR
Height (m)	166	162–173	167	162–175
Weight (kg)	59.8	53.8–67.5	60.7	54.4–68.1
BMI (kg/m^2^)^b^	22.0	3.9	22.1	3.9
Vitamin D intake (μg/day)^c^	2.9	1.6–4.9	3.0	1.7–5.0
Calcium intake (mg/day)	767	572–1003	770	575–1013

S-25(OH)D, serum 25-hydroxyvitamin D; BMI, body mass index; IQR, inter-quartile range i.e., 25–75th percentile

No significant differences (*P* > 0.05, in all cases) were evident between the two groups

^a^Extended winter refers to sampling during November-March, and extended summer refers to sampling during April–October

^b^Values reported as Means and Standard Deviations, as normally distributed

^c^Total vitamin D intake representing that coming from diet and supplement use

### Vitamin D and calcium intakes, and their key food sources

The mean, standard deviation, median and 5th and 95th percentiles of usual vitamin D and calcium intakes (from food and supplements) in the entire sample of 428 teenagers were 3.7, 3.0, 2.9, 0.6 and 9.5 μg and 812, 331, 767, 357 and 1426 mg, respectively. Boys had significantly higher median intakes of vitamin D (+ 0.73 μg) and calcium (+ 299 mg) compared to girls (*P* < 0.001, in both cases). Additionally, 16–18-year-olds had significantly higher median intakes of vitamin D (+ 0.78 μg) and calcium (+ 58 mg) compared to 13–15-year-olds (*P* < 0.001, in both cases).

The main sources of vitamin D and calcium in the entire sample of 428 teenagers within the NTFS II are shown in Table 2. Meat and meat products, breakfast cereals, eggs and egg dishes, milk and yogurt, and fish and fish dishes were the main food-group contributors to vitamin D intake, whereas milk and yogurt, bread and rolls, grains, rice, pasta and savouries, cheeses, and meat and meat products were the main food-group contributors to calcium intake.

**Table 2:** Contribution (µg/mg and %) of food groups to mean daily vitamin D and calcium intakes in Irish teenagers aged 13–18 years

	Vitamin D		Calcium	
	µg/d	%	mg/d	%
Meat and meat products	0.7	29.2	50.8	7.2
Breakfast cereals	0.8	18.7	47.2	5.2
Eggs and egg dishes	0.4	11.2	8.8	1.1
Milk and yogurt	0.4	10.8	252.5	27.2
Fish and fish dishes	0.3	7.2	5.9	0.8
Grains, rice, pasta and savouries	0.1	4.6	83.9	10.7
Nutritional supplements	0.7	4.6	5.6	0.7
Butter and spreading fats	0.1	3.6	0.9	0.1
Creams, ice–creams and chilled desserts	0.1	2.4	21.2	2.8
Biscuits, cakes and pastries	0.0	1.8	26.8	3.6
Bread and rolls	0.0	1.5	126.9	16.9
Cheeses	0.0	1.3	74.0	8.4
Beverages	0.0	0.9	13.1	1.8
Sugars, confectionery, preserves and savoury snacks	0.0	0.9	37.8	5.1
Soups and sauces	0.0	0.5	10.2	1.3
Potatoes and potato products	0.0	0.5	12.0	1.8
Vegetables and vegetable dishes	0.0	0.1	19.8	2.9
Fruit and fruit juices	0.0	0.1	14.8	2.0
Nuts, seeds, herbs and spices	0.0	0.0	2.2	0.3

Sixty-seven and 94% of Irish teenagers within the NTFS II had intakes of calcium and vitamin D below the respective US-based EAR values [7], reflecting a high degree of inadequacy of intake of both micronutrients. There was a significant sex difference in the proportion of boys and girls with an intake of vitamin D and calcium below the respective EAR values. While 92.0% of boys had vitamin D intakes < EAR, 96.2% of girls had intakes < EAR (*P* < 0.001). In the case of calcium, 48% of boys had intakes < EAR, whereas 88% of girls had intakes < EAR (*P* < 0.001). Using EFSA’s EAR value of 960 mg/day (12–17 years) and 860 mg/day (18 years) [14], 51% of Irish teenagers within the NTFS II had inadequate intakes of calcium [7]. Again, there was a significant sex difference (*P* < 0.001) where 30% of boys and 75% of girls had calcium intakes < EAR. EFSA did not establish an EAR for vitamin D [10].

None of the teenagers within the NTFS II had vitamin D intakes above the US or European ULs [7, 31], and only one teenage participant (a 15-year-old-boy) out of the entire sample had calcium intakes above the IOM’s UL for calcium [7].

### Serum 25-hydroxyvitamin D status

The mean, standard deviation and selected percentiles within the distribution of standardized serum 25(OH)D concentrations throughout the year in the subset of 246 teenagers who provided a blood sample within the NTFS II, and by sex and age-group (13–15 years *v*. 16–18 years), separately, are shown in Table 3. Two-way ANOVA showed that serum 25(OH)D concentration was significantly affected by sex (*P* = 0.013) and age-group (*P* = 0.015), but there was no significant interaction between these two factors (*P* = 0.11). Girls had a lower mean year-round serum 25(OH)D concentration (by − 7.2 nmol/L, on average) compared with boys (*P* = 0.013), and 13–15-year-olds had a higher mean year-round serum 25(OH)D concentration (by 6.5 nmol/L, on average) compared with 16–18-year-olds (*P* = 0.015) (Table 3).

**Table 3:** Standardized serum 25-hydroxyvitamin D [25(OH)D] concentrations throughout the year in healthy teenagers in Ireland

	All subjects	All boys	All girls	All 13–15 y-olds	All 16–18 y-olds
*n*	246	123	123	119	127
Serum 25(OH)D (nmol/L)					
Mean^a^	47.8	51.5	44.3	51.3	44.7
Standard deviation	22.8	25.3	19.6	24.5	20.8
Median	45.2	49.1	43.0	50.6	42.0
25–75th Percentile	31.6–60.4	34.5–64.2	28.3–56.3	34.7–62.0	27.3–58.9
5th Percentile	18.5	20.2	14.0	23.5	14.4
95th Percentile	82.3	90.2	79.2	85.8	80.2

Data weighted for social class weights arising from comparison with national population

^a^Two-way ANOVA showed that serum 25(OH)D concentration was significantly affected by sex (*P* = 0.013; boys > girls) and age-group (*P* = 0.015; 13–15 y > 16–18 y), but there was no significant interaction between these two factors (*P* = 0.11)

Three-way ANOVA showed that serum 25(OH)D concentration was significantly affected by season (*P* = 0.017), sex (*P* = 0.013), and borderline by age-group (*P* = 0.064), but there were no significant interactions between these three factors (*P* > 0.11, in all cases). Mean (± SD) serum 25(OH)D concentration was lower in extended winter than extended summer (44.6 ± 22.7 (*n* 147) *v*. 52.2 ± 22.3 nmol/L (*n* 99), respectively). Median (IQR) daily vitamin D intakes from all sources in subjects sampled during extended summer and extended winter were 2.5 (1.5–3.9) and 3.1 (1.6–5.3) μg/d, respectively (*P* = 0.068), and were 2.2 (1.5–3.6) and 3.0 (1.5–4.5) μg/d, respectively (*P* = 0.063) using food sources only. There was no difference in the percentage supplement use among participants sampled in the two seasons (7.1% versus 8.9% for extended summer and extended winter, respectively; *P* = 0.61), and the median (IQR) vitamin D content of the supplements taken during these periods were 5 (5.0–5.0) and 10 (4.4–25.0) μg, respectively.

### Prevalence of vitamin D deficiency and inadequacy

The prevalence of teenagers within NTFS II with serum 25(OH)D < 30 nmol/L as well as between 30 and 49.9 nmol/L, and stratified by sampling period, are shown in Table 4. Within NTFS II, 21.7 and 33.1% of teenagers had serum 25(OH)D < 30 nmol/L and between 30 and 49.9 nmol/L, respectively, concentrations associated with vitamin D deficiency and inadequacy, respectively [7]. The prevalence of vitamin D deficiency was higher during extended winter (27.2%) and much lower during extended summer (14.1%), whereas there was less seasonal variation in the prevalence of vitamin D inadequacy (Table 4).

**Table 4:** The prevalence of standardized serum 25-hydroxyvitamin D [25(OH)D] concentrations < 30 nmol/L and between 30 and 49.9 nmol/L for healthy teenagers in Ireland, stratified by sampling period

Group	*n*	% Serum 25(OH)D < 30 nmol/L			% Serum 25(OH)D 30–49.9 nmol/L		
		Yearly	Extended winter	Extended Summer	Yearly	Extended winter	Extended Summer
All	246	21.7	27.2	14.1	33.1	35.5	29.8
All girls	123	26.0	37.1	15.7	32.3	29.0	35.3
All boys	123	17.3	20.0	11.3	34.0	40.3	20.3
All 13–15 y-olds	119	14.1	15.2	13.3	33.3	41.9	26.8
All 16–18 y-olds	127	28.4	33.6	15.5	33.0	32.2	35.1

Data weighted for social class weights arising from comparison with national population

In addition, 14.4% of teenagers had serum 25(OH)D < 25 nmol/L, which is another internationally suggested threshold of vitamin D deficiency [38], while 45.1% of teenagers had serum concentrations greater than 50 nmol/L, associated with adequacy of vitamin D status [7, 10]. Only two participants (0.9%) had serum 25(OH)D concentrations > 125 nmol/L (161 and 167 nmol/L, respectively), and both participants were boys in the 13–15 years age-group, with one sampled in extended summer (no supplement use) and the other sampled in extended winter (and took a supplement containing 50 μg vitamin D). The IOM suggest that there may be reason for concern at serum 25(OH)D levels above 125 nmol/L, especially if sustained [7].

The prevalence of serum 25(OH)D concentrations < 30 and in the range 30–49.9 nmol/L stratified by sex and age-group are shown in Table 4. In addition, 12.0 and 48.7% of teenage boys had serum 25(OH)D concentrations < 25 and > 50 nmol/L, respectively, whereas for teenage girls the prevalence was 16.8 and 41.7%, respectively. By age-group, 9.2 and 52.6% of 13–15 y-old teenagers had serum 25(OH)D concentrations < 25 and > 50 nmol/L, respectively, whereas for 16–18-years-old teenagers the prevalence was 19.1and 38.6%, respectively.

The percentage of all teenagers with serum 25(OH)D concentrations < 30 nmol/L was higher among non-supplement users than supplement users (21.3% versus 0%, respectively; *P* = 0.022). There was no difference in the percentage with serum 25(OH)D concentrations between 30 and 49.9 nmol/L (34.1% versus 35.0% among non-supplement users versus supplement users, respectively; *P* > 0.93).

There were stepwise increments in median (IQR) daily vitamin D intakes in subjects with serum 25(OH)D concentrations < 30, 30–50, and > 50 nmol/L at 1.9 (1.1–3.0), 3.1 (1.4–4.1) and 3.5 (2.0–6.2) μg/day, respectively (*P* < 0.001). Limiting the analysis to only those subjects sampled during extended winter also showed stepwise increments in median (IQR) daily vitamin D intakes in subjects with serum 25(OH)D concentrations < 30, 30–50, and > 50 nmol/L at 2.2 (1.1–3.0), 3.2 (1.7–4.6) and 4.8 (2.4–8.9) μg/day, respectively (*P* < 0.001). Median (IQR) daily vitamin D intakes in subjects with serum 25(OH)D concentrations < 30 and ≥ 30 nmol/L during extended summer were 1.6 (0.7–3.5) and 2.7 (1.6–4.1) μg/d, respectively (*P* = 0.047).

### Determinants of serum 25-hydroxyvitamin D and predictors of vitamin D deficiency

Potential determinants of serum 25(OH)D concentrations throughout the year in the teenagers within the NTFS II were assessed using multiple linear regression analysis. Age-group, sex, time spent outdoors, and calcium intake were non-significant (*P* > 0.06, in all cases) determinants of serum 25(OH)D concentrations. Being sampled during the extended summer (April–October) (B 8.433, SE 2.626; *P* = 0.002) and increasing usual vitamin D intake (B 1.510, SE 0.207; *P* < 0.001) were positively associated with serum 25(OH)D concentrations, whereas increasing BMI (B − 0.904, SE 0.296; *P* = 0.021), not going on a sun holiday (B − 12.028, SE 3.371, *P* < 0.001) and not being of white skin (B -15.247, SE 3.199, *P* < 0.001) were negative predictors of serum 25(OH)D concentrations. Inclusion of use of vitamin D-containing supplements and vitamin D intake from diet only as variables within the model in place of vitamin D intake from all sources (i.e., diet and supplements) showed that both were positively associated with serum 25(OH)D concentrations (B 16.393, SE 4.469; *P* < 0.001 and B 1.478, SE 0.496; *P* = 0.003, respectively) (data not shown).

Limiting the analysis to only those subjects sampled during extended winter showed that usual vitamin D intake (*P* < 0.001), a sun holiday (*P* = 0.001) and being of non-white skin colour (*P* = 0.021) were the only three significant determinants of serum 25(OH)D concentrations (data not shown). Inclusion use of vitamin D-containing supplements and vitamin D intake from diet only in place of vitamin D intake from all sources showed that both were positively associated with serum 25(OH)D concentrations (*P* = 0.006 and *P* = 0.002, respectively) (data not shown).

Limiting the analysis to only those subjects sampled during extended summer showed that usual vitamin D intake (*P* < 0.01) (or vitamin D supplement use; *P* = 0.011, but not vitamin D intake from diet only; *P* = 0.18), BMI (*P* = 0.011), sex (*P* < 0.01) and being of non-white skin colour (*P* < 0.001)) were significant determinants of serum 25(OH)D concentrations (data not shown).

Multiple logistic regression analysis was used to identify the factors contributing to serum 25(OH)D concentrations < 30 nmol/L (vitamin D deficiency) within the NTFS II teenagers (Table 5). Extended winter sampling, low usual vitamin D intake, being overweight/obese or being of non-white skin type were significant predictors of risk of vitamin D deficiency (Table 5). Sex, age-group, taking a sun holiday, and calcium intake below the median were not significant (*P* > 0.07, in all cases) predictors of risk of vitamin D deficiency (data not shown).

**Table 5:** Multiple logistic regression analysis of predictors of vitamin D deficiency (serum 25-hydroxyvitamin D < 30 nmol/L) in Irish teenagers

	OR	95% CI	*P*-value
Time of year			
April–October (extended summer)	1.00	–	–
November–March (extended winter)	2.54	1.10, 5.87	0.029
Usual vitamin D intake^a^			
Greater than median (> 2.9 μg/day)	1.00	–	–
Less than median (≤ 2.9 μg/day)	4.19	1.87, 9.39	0.001
Overweight or obese^b^			
Yes	1.00	–	–
No	0.43	0.20, 0.93	0.032
Skin colour			
Non-white	1.00	–	–
White	0.18	0.08, 0.42	0.0001

*OR* odds ratio, *95% CI* 95% confidence interval

Based on weighted data for social class weights

^a^Total vitamin D intake representing that coming from diet and supplement use

^b^Based on the International Obesity Task Force (IOTF) age-and-sex-specific BMI cut-offs for defining overweight and obesity between 2 and 18 years [33]

For those subjects sampled during extended winter, a usual vitamin D intake below the median of 2.9 μg/d (OR 4.973, 95% CI 1.926, 12.840; *P* = 0.001) was a significant predictor of vitamin D deficiency, while being aged 13–15 years reduced risk of deficiency (OR 0.326, 95% CI 0.115, 0.924; *P* = 0.035), after adjusting for possible confounders (data now shown). Limiting the analysis to only those sampled during extended summer showed that not being overweight/obese (OR 0.101, 95% CI 0.017, 0.619; *P* = 0.013) and being of white skin type (OR 0.020, 95% CI 0.002, 0.187; *P* = 0.001) significantly lowered risk of vitamin D deficiency, after adjusting for possible confounders (data now shown).

## Discussion

Adolescence is a nutrition-sensitive phase of growth and a transformative life phase that can have profound consequences on an individual’s health in later life [39]. The vast majority of adult bone mass is accrued throughout adolescence [40]. For example, it has been estimated that during adolescence, approximately 40% of total bone mineral is acquired during the 4 years surrounding the peak in bone accretion in the early teenage years and that 95% of adult bone mass has been achieved by 4 years following this peak [41]. As part of the evidence grading scheme, the NOF have highlighted the importance of calcium and vitamin D as modifiable lifestyle factors for the development of PBM [5]. However, calcium and vitamin D have been identified as short-fall nutrients of public health concern in the last three successive US reports on *Dietary Guidelines for Americans*, spanning a period 2010–2025 [42–44]. Of concern, the present analyses showed that calcium intakes were inadequate in between half to two-thirds of Irish teenagers aged 13–18 years, depending on whether the US EAR or the EU equivalent value [7, 14] was used as the benchmark to assess adequacy. Moreover, there was evidence of sex differences, whereby 30–48% of teenage boys and 75–88% of teenage girls had inadequate intakes of calcium. The lower calcium intakes in girls are likely related to less food intake overall, as reflected by their lower total calorie intake compared to that of boys, but also lower intakes of dairy foods as well as breads [20], which are key contributory food sources to calcium intakes in Ireland. The prevalence of calcium-containing supplement use was also low in both groups (2 and 4% for boys and girls, respectively). The US Tolerable Upper Intake Levels for calcium in teenagers aged 13–18 years is 3000 mg/day [7]. The 95th percentile of usual calcium intake in the entire sample of teenagers was 1426 mg/day (1565 and 1136 mg/d for boys and girls, respectively), suggesting there was room for improvement in calcium intakes, be it by natural foods, fortified foods and/or supplements as the strategies highlighted by the FAO-WHO in terms of addressing inadequacy of micronutrient intake [45]. As emphasised recently, such strategies need to be cognizant of widely shared adolescent values beyond nutrition and health [46].

In terms of assessment of population vitamin D nutriture, serum 25(OH)D concentrations (reflecting the contributions from both diet and dermal synthesis [47]) needs to be considered together with dietary intake data for the vitamin [7]. The IOM and other agencies/expert groups have used serum 25(OH)D concentrations below a threshold of 30 nmol/L as being indicative of increased risk of vitamin D deficiency [7, 10, 12], as reflected by impaired fractional calcium absorption, lower bone mineral content and/or density as well as increased risk of rickets/osteomalacia [7]. In the present study, 21.7% of a representative sample of 13–18-year-old teenagers in Ireland (51–54 °N) had serum 25(OH)D concentrations < 30 nmol/L across the year, and the prevalence of vitamin D deficiency was higher (27.2%) in subjects sampled during extended winter (November–March). There are only a limited number of other studies of vitamin D status in Europe that have used a nationally representative sample for this age-group [19, 48, 49], but importantly the prevalence estimates from most of these have been based on standardized serum 25(OH)D values allowing for more valid comparisons with the current study. Data from the *Young Hearts 2000* (YH2000) cohort of 12 and 15 year-old adolescents in Northern Ireland (53-55^o^N) suggest a prevalence of 26% with serum 25(OH)D < 30 nmol/L [48]. However, it should be noted that no sampling took place during July or August in either that study or the present study because of school holidays. Recent data from the NDNS rolling programme, covering the period 2008/9–2018/19, showed that 15–26% of 11–18-year-old adolescents in the UK (latitude range of 50–59 °N) had serum 25(OH)D < 25 nmol/L, depending on sampling years [19]. The *German Health Interview and Examination Survey for Children and Adolescents* (KiGGS), conducted from 2003 to 2006 (latitude range of 47–55 °N), showed that 13.9 and 18.3% of female and male adolescents (aged 14–17 years), respectively, had serum 25(OH)D < 30 nmol/L [49]. The population-based, multi-centred *Healthy Lifestyle in Europe by Nutrition in Adolescence* (HELENA) cross-sectional study of adolescents aged 12.5–17.5 years, conducted from 2005 to 2008 in ten European cities (latitude range of 35–59 °N), showed that 12.2% had serum 25(OH)D < 30 nmol/L [37].

Circulating 25(OH)D concentrations greater than 30 nmol/L but less than 50 nmol/L may reflect inadequacy of vitamin D status [7]. About one-third (33.1%) of teenagers in the present study had 25(OH)D concentrations in the range 30–49.9 nmol/L across the year. These findings are broadly in line with those of the three previously mentioned studies of representative samples of German, UK and European adolescents (32%, 35%, and 37%, respectively) [19, 37, 49]. The importance of comparison of prevalence estimates for vitamin D deficiency and inadequacy at broadly similar latitudes, such as the above-mentioned German and UK surveys, is exemplified by a comparison of the present prevalence estimates from NFTS II with those from the *National Health and Nutrition Examination Survey* (NHANES) study in the US (latitude range of 25–47 °N) which showed that only 4.7% and 22.7% of 12–19-year-olds had standardized serum 25(OH)D < 30 and 30–49 nmol/L, respectively [50]. Likewise for other regions of the World at lower latitudes, such as parts of central America and Asia, generally the prevalence of serum 25(OH)D < 30 nmol/L in national surveys is < 8%, albeit not based on standardized data [16].

In the current study, teenage girls had lower mean serum 25(OH)D levels than boys of the same age. There was an increased proportion of girls with serum 25(OH)D concentrations < 30 nmol/L throughout the year (26.0% v. 17.3%, for girls and boys, respectively). Likewise, data from the NDNS rolling programme showed that the prevalence of serum 25(OH)D < 25 nmol/L in girls aged 11–18 years was in the range 15–39%, depending on sampling years (mean, 21.0%), whereas it was 12–21% (mean, 16.1%) for equivalently aged boys [19]. The Northern Ireland YH2000 study found that 15-year-old boys had significantly higher serum 25(OH)D concentrations than girls, although there was no significant sex-related difference observed in serum 25(OH)D concentration in the 13-year-old teenagers [48]. In Germany, data on 14–17-year-adolescents from KiGGS showed that girls had a lower prevalence of serum 25(OH)D < 30 nmol/L than boys (13.9 and 18.3%, respectively), although this trend was reversed in the case of adolescents aged 11–13 years (18.9 *v*. 11% for girls and boys, respectively) [49]. There have also been mixed findings in relation to potential sex differences in prevalence of vitamin D deficiency in national surveys outside of Europe [16].

The reason for the apparent sex-related difference in the present study is unclear. There was a greater proportion of boys than girls (68% *v*. 48%, respectively) sampled in the darker part of the year (November to March) which might have been expected to inflate the prevalence of serum 25(OH)D < 30 nmol/L across the year in boys. However, even within the extended winter period, the higher prevalence amongst girls was still evident (37.1% *v*. 20.0%, for girls and boys, respectively), while in the extended summer the deviation became less (15.7% *v*. 11.3, for girls and boys, respectively). In the current study, girls had lower vitamin D intakes than boys and this may have been of importance, particularly in winter-time when dermal synthesis of vitamin D would have been largely absent. It is also interesting to note that subjects, irrespective of sex, with vitamin D deficiency and inadequacy had significantly lower vitamin D intakes compared with those in vitamin D-replete subjects. Multiple linear regression analysis of subjects sampled in extended winter showed that vitamin D intake, whether included in the model as intake from all sources or as vitamin D supplement use and separately vitamin D intake from food sources only, as well as taking a sun holiday and skin colour, were significant determinants of serum 25(OH)D levels. In extended summer, vitamin D intake from all sources or as vitamin D supplement use were also significant, albeit weaker, determinants of serum 25(OH)D levels, together with sex, BMI, and skin colour. Vitamin D intake from food sources only was not a significant determinant in summer.

To explore the reasons for the high prevalence of vitamin D deficiency in the adolescents in the Republic of Ireland, we investigated the potential predictors of serum 25(OH)D < 30 nmol/L using multiple logistic regression analysis. Not surprisingly, season and low vitamin D intake were the major determinants of vitamin D status, a finding shown in studies of adolescents elsewhere [48, 50–52] as well as in Irish adults [53]. As season was such a major predictor, we repeated the analysis in the subgroups sampled during extended winter- and extended summertime separately. During extended winter, being aged 16–18 years and low vitamin D intake (< 2.9 μg/day) were significant predictors of having a serum 25(OH)D value of < 30 nmol/L. That vitamin D intakes below the median of 2.9 μg/day for the present group of teenagers predicted vitamin D deficiency aligns with data on the dietary vitamin D requirement of UK adolescents aged 14–18 years in which a vitamin D intake during wintertime of 3.0 μg/day only allowed 50% of adolescents achieve a serum 25(OH)D of > 30 nmol/L, and an intake of 10.9 and 13.1 μg/day was needed to allow 95 and 97.5% of adolescents, respectively, achieve this wintertime serum 25(OH)D threshold [54]. This is of concern considering 94% of the teenagers in the present study failed to meet the US EAR for vitamin D of 10 μg/day [7].

The low intakes of vitamin D in Irish teenagers are in line with similar reports from several other European countries with national nutrition surveys [15]. For example, data from the rolling NDNS (2008/9–2018/19) showed that the mean and median daily intake of vitamin D from all sources (including nutritional supplements) for adolescents (aged 11–18 years) in the UK was 2.8 and 2.0 μg, respectively [19]. The mean vitamin D intake of children aged > 10 years in 14 European member states ranged from approximately 1 to 4 μg/day, with an average for Europe of 2.2 μg/day [15, 16]. Similarly, the mean vitamin D intake of adolescents aged 12.5–17.5 years participating in the HELENA cross-sectional study of 10 European cities was 1.8 μg/day [55]. For teenagers living in Ireland, the UK and elsewhere in Europe, low intakes of vitamin D may take on increased importance during wintertime when sunlight is of insufficient intensity to stimulate dermal vitamin D synthesis. The finding that the older teenage age-group (16–18 years) was a significant predictor of risk of vitamin D deficiency may have been linked with advanced pubertal status. In a study of US-based school-age children, those in late stages of puberty (Tanner 4 and 5; mean age 13.7 years) had significantly lower mean serum 25(OH)D than prepubertal children (Tanner 1; mean age 9.0 y) [56]. Time spent in physical activities was similar in the two teenage age-groups in the present survey [20], but this unfortunately was not delineated into time in outdoor physical activities, which would have been of value for the present analyses. There may also be other reasons underpinning the age-related differences in status.

EFSA in setting their Adequate Intake value for vitamin D of 15 mg/day, assumed conditions of minimal cutaneous vitamin D synthesis [10], as did the IOM when establishing their recommended intakes [7]. They also state that in the presence of cutaneous vitamin D synthesis, the requirement for dietary vitamin D is lower or may even be zero [10]. This underscores the need to consider dietary intake data together with serum 25(OH)D concentrations, where feasible, as mentioned above. The impact of UVB-derived vitamin D supply is evident in the data on prevalence of serum 25(OH)D < 30 nmol/L in the present survey, with the estimate from extended summer being about half that from extended summer, despite broadly similar vitamin D intakes. However, it was still notable that 14% of adolescents living in Ireland had serum 25(OH)D < 30 nmol/L during extended summertime, at a time when vitamin D synthesis and status would be expected to be optimum. Thus, an understanding of predictors of such low vitamin D status during summer could be important in terms of preventative strategies. Vitamin D intakes in those with serum 25(OH)D concentrations < 30 nmol/L were significantly lower than in those with concentrations ≥ 30 nmol/L during extended summer. The present work also highlighted how being overweight or obese increased risk of vitamin D deficiency among the Irish teenagers during summer. This is a concern in light of the fact that the prevalence of combined overweight and obese within the NTFS II (2019–2020) sample at 23.6% has increased from the 18% observed within the NTFS in 2005–2006 [17, 20]. In the US, data for 12–19-year-old female participants in the US NHANES III shows that those who were overweight had a 75% increased risk of serum 25(OH)D concentrations < 50 nmol/L compared to adolescents within the normal weight/BMI category [57]. In a nationally representative survey in Australia, overweight adolescents (aged 12–17-year-old) were approximately twice as likely to have serum 25(OH)D < 50 nmol/L compared to underweight/healthy weight adolescents [58]. The present work also highlighted how being of white skin colour reduced risk of serum 25(OH)D of < 30 nmol/L during summer compared to being of darker skin colour. This aligns with international evidence for elevated risk of low vitamin D status among dark-skinned ethnic subgroups of the population [37, 50]. Increased skin pigment can greatly reduce the UVB radiation-mediated synthesis of vitamin D [59]. Also to note that emerging evidence suggests a higher dietary requirement for vitamin D to maintain serum 25(OH)D > 30 nmol/L in dark-skinned individuals compared to white residing at higher latitudes [60].

The clear strengths of the present work include the nationally representative sample of teenagers, the standardized serum 25(OH)D data as well as the use of statistical modelling to estimate usual intakes accounting for day-to-day intra-person variation resulting in a better estimate of the true distribution of nutrient intakes. The modelling also improves the estimates of the proportions of the population with intakes above or below particular reference values (e.g. UL, EAR) which otherwise would be overestimated. However, it has some limitations also. In terms of the sampling distribution throughout the year with a higher proportion of teenagers sampled in the December to February wave (47%) than the other three seasonal waves (17–20%). Provision of blood samples in summer months was logistically more challenging for some participants. The survey did not capture information on the pubertal status of the teenagers which may have shed additional light on why the 16–18-year-old teenagers had lower vitamin D status that those aged 13–15 years. Assessment of pubertal status is not currently part of the methodology of the nutrition surveys of teenagers in Ireland.

In conclusion, there was a high prevalence of inadequacy of intake of calcium and vitamin D in Irish teenagers. In addition, a fifth of teenagers had serum 25(OH)D < 30 nmol/L, reflective of increased risk of vitamin D deficiency. In light of the importance of adequate calcium and vitamin D for bone health during the critical adolescent period, increased emphasis needs to be given to exploring strategies for improving the intake of both bone-related micronutrients in adolescents. Such work would help address the recently highlighted underinvestment in research on nutrition during adolescence as compared with research in other age groups, which has inhibited the development of adolescent-responsive nutritional policies [39].

## Data Availability

None.

## Abbreviations

25(OH)D: 25-Hydroxyvitamin D
ANOVA: Analysis of variance
BMI: Bone mass index
EAR: Estimated average requirement
EFSA: The European Food Safety Authority
IOM: Institute of Medicine
IQR: Interquartile range
IUNA: Irish Universities Nutrition Alliance
MDI: Mean daily intake
NDNS: National Diet and Nutrition Survey
NHANES: National Health and Nutrition Examination Survey
NTFS-II: National Teens Food Survey II
OR: Odds ratio
PBM: Peak bone mass
UL: Tolerable Upper Intake Level
YH2000: Young Hearts 2000 cohort study
